# Generation and characterization of a knockout mouse of an enhancer of *EBF3*

**DOI:** 10.1242/bio.062070

**Published:** 2025-11-07

**Authors:** Emily Cordova Hurtado, Janine M. Wotton, Alexander Gulka, Crystal Burke, Jeffrey K. Ng, Ibrahim Bah, Juana Manuel, Hillary Heins, Stephen A. Murray, David U. Gorkin, Jacqueline K. White, Kevin A. Peterson, Tychele N. Turner

**Affiliations:** ^1^ Department of Genetics, Washington University School of Medicine, St. Louis, MO 63110, USA; ^2^ The Jackson Laboratory, Bar Harbor, ME 04609, USA; ^3^ Department of Biology, Emory University, Atlanta, GA 30322, USA

**Keywords:** Enhancer, Regulatory, Mouse, Knockout, Brain

## Abstract

Genomic studies of neurodevelopmental disorders (NDDs) have identified several relevant genomic variants. *EBF3* is a gene with an excess of protein-coding *de novo* variants and underlies Hypotonia, Ataxia, and Delayed Development Syndrome. We previously identified noncoding *de novo* variants in an enhancer of *EBF3* and further found enrichment of deletions of this enhancer in NDDs. In this study, we generated a novel mouse line that deletes the highly conserved, orthologous mouse region within the Rr169617 regulatory region, and characterized the molecular and phenotypic aspects of this mouse model. We found a deviation from Mendelian expectation (*P*=0.02) with significant depletion of the deletion allele (*P*=5.8×10^−4^). *Rr169617^+/−^* mice had a reduction of *Ebf3* expression by 10% and *Rr169617^−/−^* mice had a reduction by 20%. Differential expression analyses in E12.5 forebrain, midbrain, and hindbrain in *Rr169617^+/+^* versus *Rr169617^−/−^* mice identified dysregulated genes including histone and brain development related genes. *A priori* phenotyping analysis (open field, hole board and light/dark transition) identified sex-specific differences in mobility only for *Rr169617^−/−^* mice across multiple behavioral assays with *Rr169617^−/−^* males less mobile than *Rr169617^−/−^* females. Furthermore, both sexes when homozygous for the enhancer deletion displayed body composition differences when compared to wildtype mice. Overall, we show that deletion within Rr169617 reduces expression of *Ebf3* and results in phenotypic outcomes consistent with potential sex specific behavioral differences.

## Teaser

**Summary:** This study focuses on the generation and initial characterization of a knockout mouse for a region of noncoding, regulatory DNA that was previously implicated in autism.

## INTRODUCTION

Autism is a neurodevelopmental disorder with high heritability (Bai et al., 2019; Sandin et al., 2017). Several studies focusing on exome sequencing have identified *de novo* variants (DNVs) that disrupt genes (De Rubeis et al., 2014; Dong et al., 2014; Iossifov et al., 2014, 2012; Sanders et al., 2012; Krumm et al., 2015; O'Roak et al., 2011, 2014, 2012a,b; De Rubeis and Buxbaum, 2015). Other genetic factors include large copy number variants (Coe et al., 2014; Cooper et al., 2011; Girirajan et al., 2013; Krumm et al., 2013, 2015; Sanders et al., 2011, 2015; Levy et al., 2011; Marshall et al., 2008; Pinto et al., 2010) and common variants contributing to polygenic risk (Weiner et al., 2017), respectively. A contribution from noncoding DNVs has also been identified from studies using whole-genome sequencing (Turner et al., 2017, 2016; An et al., 2018; Werling et al., 2018; Brandler et al., 2018, 2016; Padhi et al., 2021; Markenscoff-Papadimitriou et al., 2020; Zhou et al., 2019). We previously identified an enhancer, hs737, with an excess of noncoding DNVs in individuals with autism (Padhi et al., 2021). This enhancer targets the gene *EBF3* that is the underlying gene for Hypotonia, Ataxia, and Delayed Development Syndrome (HADDS). Protein-coding DNVs of *EBF3* are also known to be genome-wide significant for excess in neurodevelopmental disorders (Chao et al., 2017; Harms et al., 2017; Sleven et al., 2017; Padhi et al., 2021; Ignatius et al., 2022). When comparing individuals with protein-coding DNVs in *EBF3* to those with noncoding DNVs in hs737, that affects *EBF3*, we found that individuals with protein-coding DNVs are more severe in their phenotype (Padhi et al., 2021). Beyond single point variants in this enhancer, we also previously showed that it does not deviate from the copy number of two in 56,256 alleles from individuals who do not have neurodevelopmental disorders (Padhi et al., 2021). However, it is enriched for deletions and nominally enriched for duplications in individuals with neurodevelopmental disorders (Padhi et al., 2021).

The *EBF3* gene encodes a transcription factor that preferentially binds to the promoters of other transcription factors and chromatin-binding proteins involved in neurodevelopmental disorders (NDDs) (e.g. *CHD2*, *CHD8*, *ARID1B*) (Padhi et al., 2021). This gene is a member of the EBF gene family, which includes EBF1, EBF2, EBF3, and EBF4 (Liberg et al., 2002), and is known to form homodimers or heterodimers with itself or other family members, respectively. It is known to be regulated by the X chromosome gene *ARX* that is also involved in NDDs. It resides in a large TAD region in the genome of ∼2 Mbp and several regulatory regions of *EBF3* exist within the TAD. The hs737 enhancer is ∼1.5 Mbp from the promoter of *EBF3* and has been shown to contact the promoter (Padhi et al., 2021; Chen et al., 2024). It is an enhancer that is a member of the VISTA enhancer database that contains several enhancers with conservation in human, mouse, and rat (Pennacchio et al., 2006). While expression of *EBF3* is ubiquitous in the human body, the activity of hs737 seems to be restricted to the fetal brain (Padhi et al., 2021).

As noted, there is an enrichment of DNVs within hs737 in individuals with autism and an enrichment of deletions in individuals with neurodevelopmental disorders. We sought to determine the molecular and phenotypic consequence of deletion of hs737 in a model system. Thus, we focused on generating a mouse model for this genomic interval as the sequence of hs737 is highly conserved with its orthologous mouse sequence [within the Rr169617 (https://www.informatics.jax.org/marker/MGI:8255218) regulatory region] (Padhi et al., 2021). Here, we describe the creation of a novel mouse line engineered to delete the relevant sequence within Rr169617, assess the molecular consequences through RNA-seq experiments, and determine the phenotypic consequences through systematic broad-based phenotyping assays. This mouse model provides a useful tool to others in the field especially those studying the *EBF3* gene regulatory network (GRN) that has been implicated in autism and other neurodevelopmental disorders (Chao et al., 2017; Harms et al., 2017; Sleven et al., 2017; Padhi et al., 2021; Ignatius et al., 2022).

## RESULTS

### The topologically associating domain with Hs737 is highly conserved in mouse

**Fig. 1.**
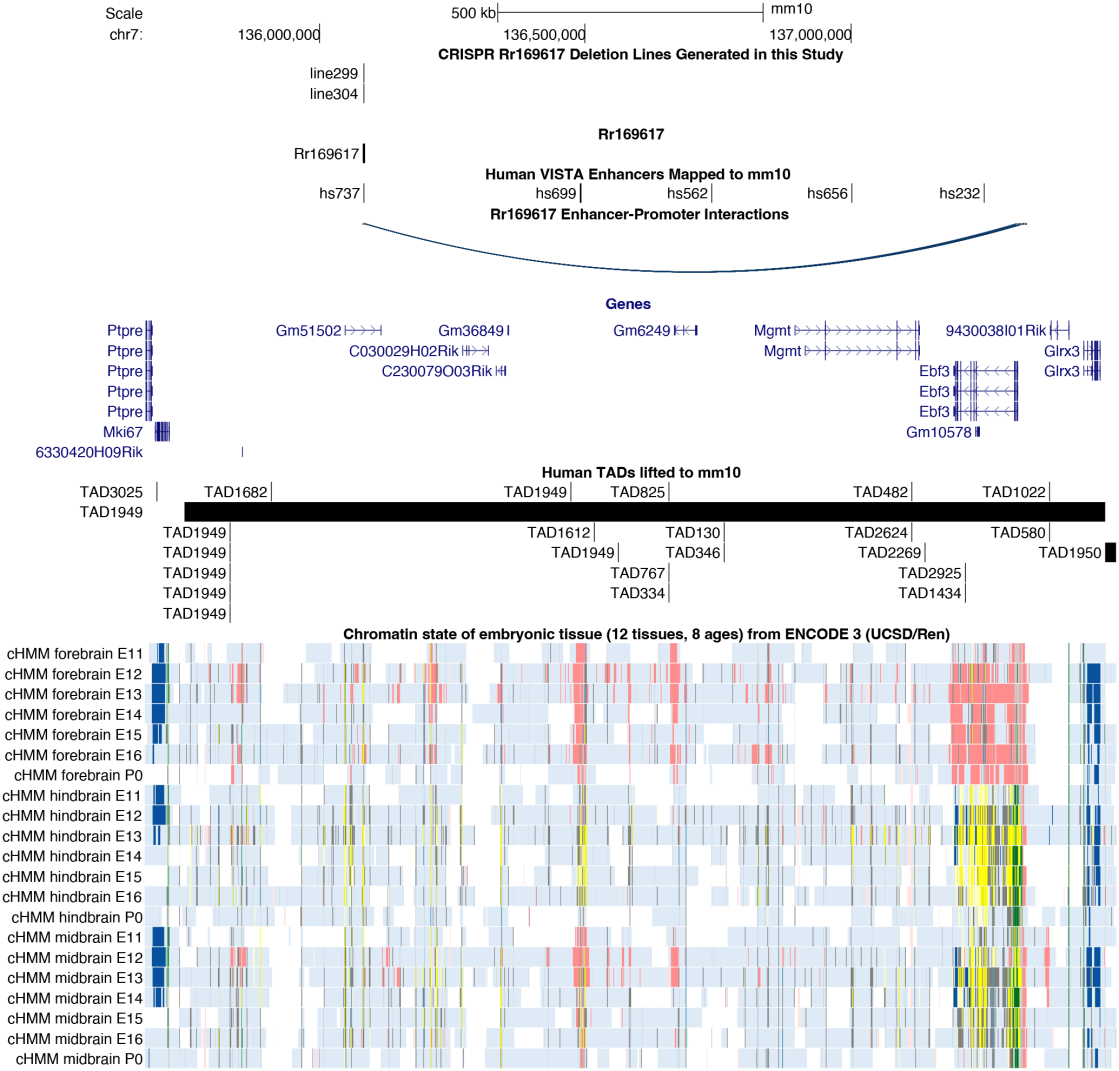
***Ebf3* regulatory landscape and associated hs737/Rr169617 enhancer deletion mouse lines.** Genome browser view of the topologically associating domain region containing Rr169617 and its target gene *Ebf3* (GRCm38/mm10). The first track shows the two independent founder mouse lines generated in this study: line 299 (C57BL/6J-Rr169617^em1Tnt^/J) and line 304 (C57BL/6J-Rr169617^em2Tnt^/J). The second track shows the location of the regulatory region, Rr169617. The third track shows the location of human VISTA enhancers lifted over to the mouse genome. Included is hs737 that resides within the Rr169617 region. The fourth track shows enhancer-promoter interactions of Rr169617 and *Ebf3* from Chen et al. (2024). The fifth track shows the genes within the region. The sixth track shows human topologically associating domains lifted over to this region and show high conservation. Finally, the chromatin state data available from ENCODE3 is shown across the different timepoints in mouse development. The colors in this track refer to their chromHMM status as described in (Ernst and Kellis, 2012).

By aggregating genomic annotation data from our (Padhi et al., 2021) and others' (Dixon et al., 2012; Chen et al., 2024; Moore et al., 2020) previous work, we studied the features of the human genomic topologically associating domain (TAD) region containing hs737 in the mouse genome (within Rr169617). *EBF3* resides within TAD1949 originally defined in Dixon et al. (2012) via Hi-C in human embryonic stem cells. To identify the orthologous region in mouse, we performed *liftover* from human (GRCh38/hg38) to mouse (GRCm38/mm10) and found that this TAD was mostly conserved in mouse (Fig. 1). The large TAD region contains the same TAD boundaries as seen in human (Fig. 1). Further support for the conserved architecture of this region is provided by comprehensive capture-Hi-C experiments for several VISTA enhancers that identified multiple enhancer-promoter interactions between the region within Rr169617 and the promoter of *Ebf3* (Fig. 1) (Chen et al., 2024) These findings are consistent with our previous observations of hs737 contacting the promoter of *EBF3* by examining Hi-C data in the human fetal brain (Chen et al., 2024; Padhi et al., 2021).

### Generation of knockout mouse lines

**Fig. 2.**
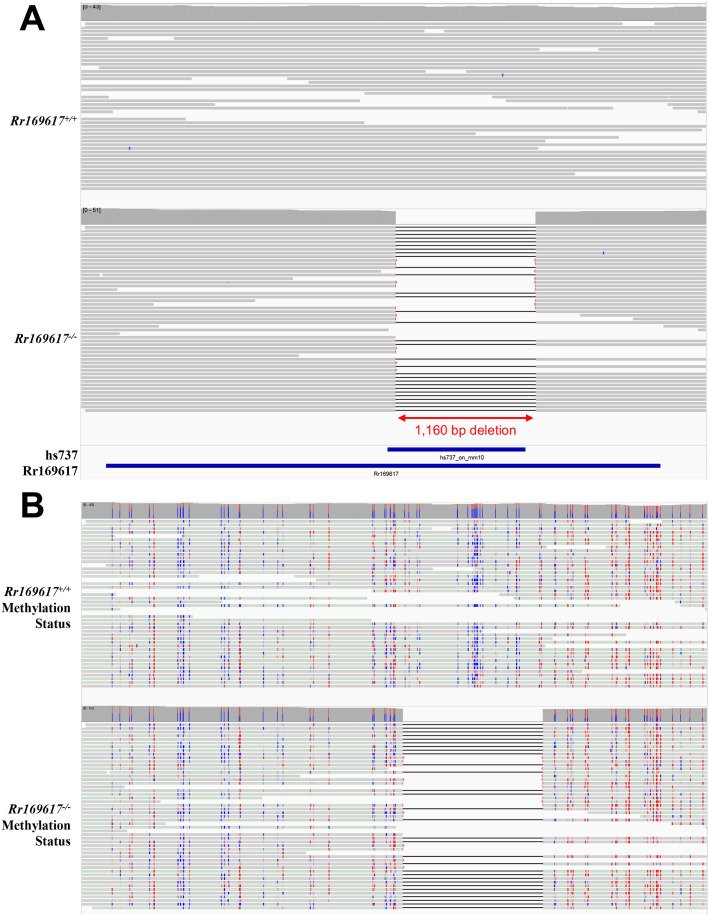
**Long-read whole genome sequencing characterization of Cas9 edited mice.** (A) PacBio HiFi long-read whole-genome sequencing of E12.5 forebrain tissue collected from *Rr169617^+/+^* and *Rr169617^−/−^* mice. All reads in the homozygous deletion mice contain the deletion. None of the reads in the wildtype mice contain the deletion. (B) Methylation status of CpG sites within the Rr169617 region based on the PacBio whole-genome sequencing data. Note, any of the bases within the portion of Rr169617 containing the sequence orthologous to hs737 are unmethylated as shown in blue. Red=methylated CpG. Blue=unmethylated CpG. For both A and B, the region shown is chr7:136,080,610-136,085,789 (GRCm38/mm10).

A deletion within Rr169617 was generated in mouse using CRISPR/Cas9 and paired guide RNAs flanking the region of interest. This resulted in two separate deletion founder animals that we carried forward (Fig. 1). Due to the similarity in the deletions, detailed experimental characterization was performed on line 299 that has a 1160 bp deletion (chr7:136083275-136084434 GRCm38/mm10). To further validate this line, we performed PacBio HiFi long-read whole-genome sequencing on E12.5 forebrain tissue from wildtype (*Rr169617^+/+^*) and homozygous deletion animals (*Rr169617^−/−^*). All of the sequence reads in the homozygous deletion (*Rr169617^−/−^*) mice contained a 1160 bp deletion and none of the sequence reads in the wildtype (*Rr169617^+/+^*) mice contained the deletion (Fig. 2A). Since we performed long-read sequencing, we also assessed the methylation (5mC) status within the enhancer region (Fig. 2B) and over the *Ebf3* promoter (Fig. S1). The sequence deleted in *Rr169617^−/−^* contained CpG sites that were not methylated in *Rr169617^+/+^*. The methylation status surrounding the deletion region was similar in both *Rr169617^+/+^* and *Rr169617^−/−^* (Fig. 2B). The *Ebf3* promoter sequence was mostly not methylated in both *Rr169617^+/+^* and *Rr169617^−/−^* (Fig. S1).

Based upon the results of long-read sequencing, we developed a PCR-based genotyping assay that could discriminate between wildtype (*Rr169617^+/+^*), heterozygote (*Rr169617^+/−^*), and homozygous deletion (*Rr169617^−/−^*) mice (Fig. S2A) and showed that it matched exactly to the results of whole-genome sequencing (Fig. S2B).

### Underrepresentation of the deletion allele

Genotype distributions were analyzed in 278 mice derived from intercrosses between heterozygous animals (*Rr169617^+/−^*×*Rr169617^+/−^*, Fig. S3). From these crosses, we observed 99 (35.6%) wildtype animals *Rr169617^+/+^*, 121 (43.5%) heterozygotes *Rr169617^+/−^*, and 58 (20.9%) homozygotes *Rr169617^−/−^*. This distribution significantly deviates from expected Mendelian frequencies (Chi-Square Test, *P*=0.02), demonstrating an underrepresentation of the deletion allele (Binomial test, *P*=5.8×10^−4^, based on an expected deletion allele frequency of 50%). This showed a confining of homozygous viability but at a reduced level with no significant differences between sexes.

### Consequence of Rr169617 deletion on Ebf3 expression

**Fig. 3.**
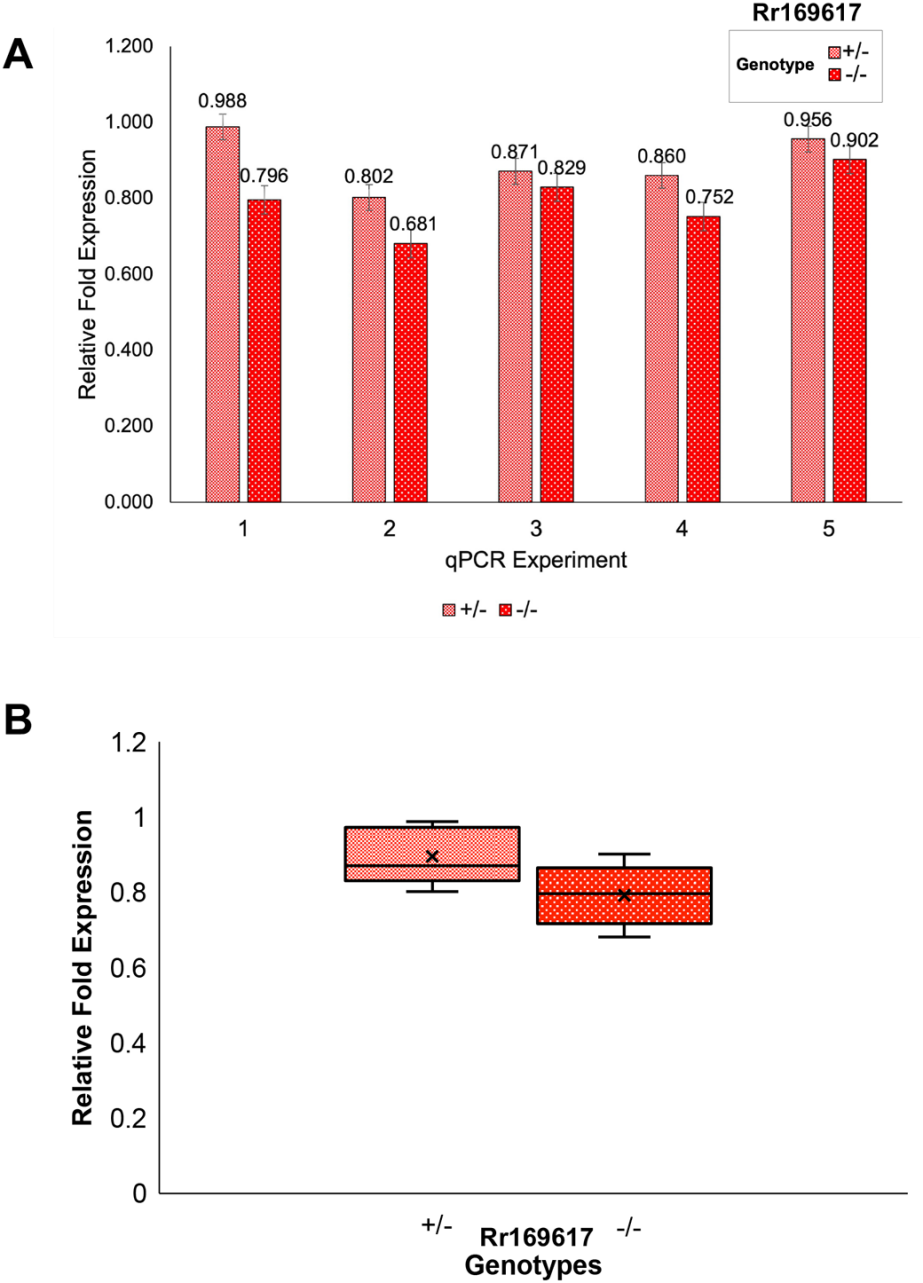
**qRT-PCR analysis for *Ebf3* expression in E12.5 forebrain.** (A) Results of five independent qRT-PCR for *Ebf3* expression. Samples for each genotype were as follows: *Rr169617^+/+^* (*n*=12), *Rr169617^+/−^* (*n*=14), and *Rr169617^−/−^* (*n*=10). (B) Relative fold expression aggregating data across all five independent qPCR experiments. For both A and B, relative fold expression is in comparison to the *Rr169617^+/+^* results.

We hypothesized that deletion in Rr169617 would affect the expression of *Ebf3* based upon the supporting 3D interaction data suggesting that it functions as an enhancer of *Ebf3*. To determine the impact of the deletion on *Ebf3* expression, we collected ≥10 mouse forebrains, of each genotype, at E12.5 and performed a series of five independent qRT-PCR experiments. Since we have previously shown that the enhancer is active at E12.5 in the forebrain (Padhi et al., 2021), we utilized this tissue source for the qRT-PCR. Consistently, the heterozygous deletion line reduced *Ebf3* expression by ∼10% (Fig. 3) compared to wildtype, and the homozygous deletion line showed a ∼20% reduction in *Ebf3* (Fig. 3). While the reduction was consistent, it was not statistically significant. For significance estimates, we calculated the sample size necessary to detect 80% power for a 20% reduction in expression and found that we need a minimum of 38 samples of each genotype, for 90% power 44 samples of each genotype, and for 100% power 68 samples of each genotype.

**Fig. 4.**
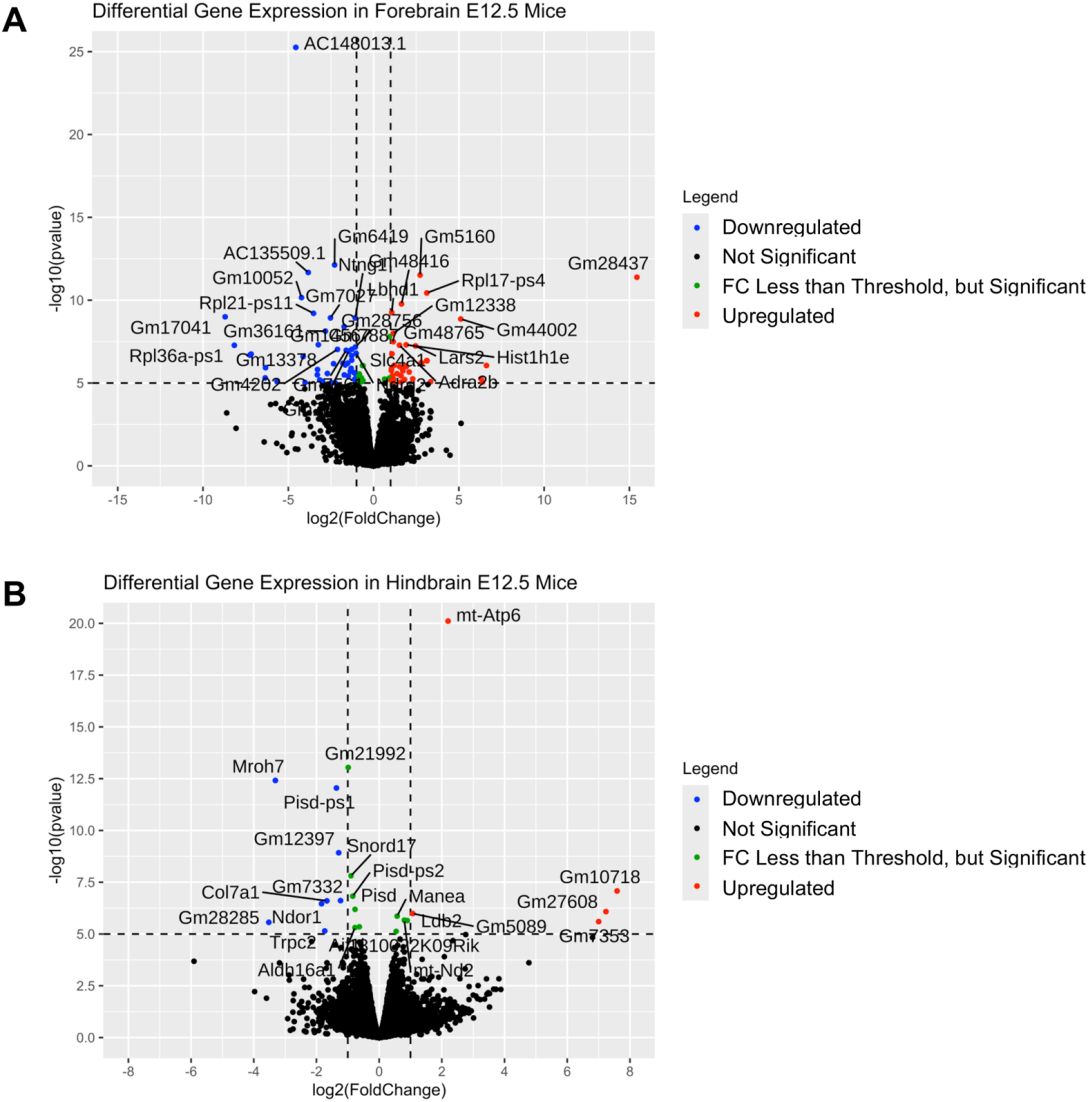
**Volcano plots of RNA-seq data from E12.5 mice.** Volcano plots from (A) forebrain and (B) hindbrain mice collected at E12.5 are shown. Each figure compares a *Rr169617^−/−^* versus *Rr169617^+/+^*. The fold change cutoff was Log2≥|1|, and a *P*-value cutoff of ≤10^−6^ was used, denoted by the dashed black lines. Red dots are significantly up-regulated genes, blue dots are significantly down-regulated genes, and green dots are genes that met statistical significance but did not meet the fold change threshold.

Based on our power estimates, we knew we would not be powered to see a significant difference in *Ebf3* expression in a standard RNA-seq experiment because it would be cost prohibitive to sequence a minimum of 76 mice [38 wildtype, 38 homozygous deletion, $737 per sample (total of $56,012) just for sequencing]. This is an important note for researchers focused on effects of variation in noncoding regions. Therefore, we proceeded to look for downstream (of *Ebf3*) expression changes of much higher effect using a high coverage (∼200 million read pairs) RNA-seq experiment on three animals of each genotype expanding out to examine the forebrain, midbrain, and hindbrain, respectively. We used a fold change cutoff of Log2≥|1| and a *P*-value cutoff of ≤10^−6^ to identify dysregulated genes. Through these analyses (Fig. 4, Fig. S4), we found there was at least one dysregulated gene in each brain region. In the forebrain (Table S1), there were 39 genes that were significantly upregulated (17 were protein-coding genes: *Lbhd1*, *Slc4a1*, *Hist1h1e*, *Adra2b*, *Lars2*, *Trim10*, *Nhej1*, *Csf2rb*, *Ncf4*, *Slc25a37*, *Prr15l*, *Acp5*, *Hist1h2bk*, *Hist1h3i*, *Mylk3*, *Cited4*, *Pdzk1ip1*) and 45 genes that were significantly downregulated (11 were protein-coding genes: *Ntng1*, *Ndrg2*, *Hist1h2ao*, *Nox1*, *Neurod6*, *Cst6*, *Cdh12*, *Chd5*, *Htra1*, *Prdm8*, *Glra2*). There were also 14 genes that were significant but did not meet the fold change threshold (11 were protein-coding genes: *Robo2*, *Cachd1*, *B4galt5*, *Bhlhe22*, *Kel*, *Hbb-y*, *Hsd3b6*, *Csf2ra*, *Cabp1*, *Asrgl1*, *Hspa8*). In the midbrain (Table S2), there was only 1 gene that was significantly downregulated (pseudogene *Pisd-ps1*). In the hindbrain (Table S3), there were 5 genes that were significantly upregulated (1 protein-coding: *mt-Atp6*) and 8 genes that were significantly downregulated (four were protein-coding genes: *Mroh7*, *Col7a1*, *Ndor1*, *Trpc2*). There were also ten genes that were significant but did not meet the fold change threshold (six were protein-coding: *Pisd*, *Manea*, *mt-Nd2*, *Ldb2*, *Aif1l*, *Aldh16a1*).

### Phenotyping analysis of enhancer deletion mice

Given the reproducible reduction in expression of *Ebf3* observed in our enhancer deletion mice, we next performed *in vivo* phenotyping (Table S4-S23) to assess potential behavioral differences focusing on open field, hole board and light/dark transition. These tests allowed us to assess traits related to anxiety, exploration, and mobility.

The PhenStat analysis revealed sexual dimorphism for the number of center entries (*P*=0.04) and total number of rears in open field (*P*=0.03). Male *Rr169617^−/−^* mice showed significantly fewer entries and less rearing compared to wildtype males, and the *Rr169617^−/−^* females showed significantly more entries and more rearing compared to wildtype females (Table S22). This indicates that female mutants explore more than female wildtypes, and male mutants explore less than male wildtypes.

**Fig. 5.**
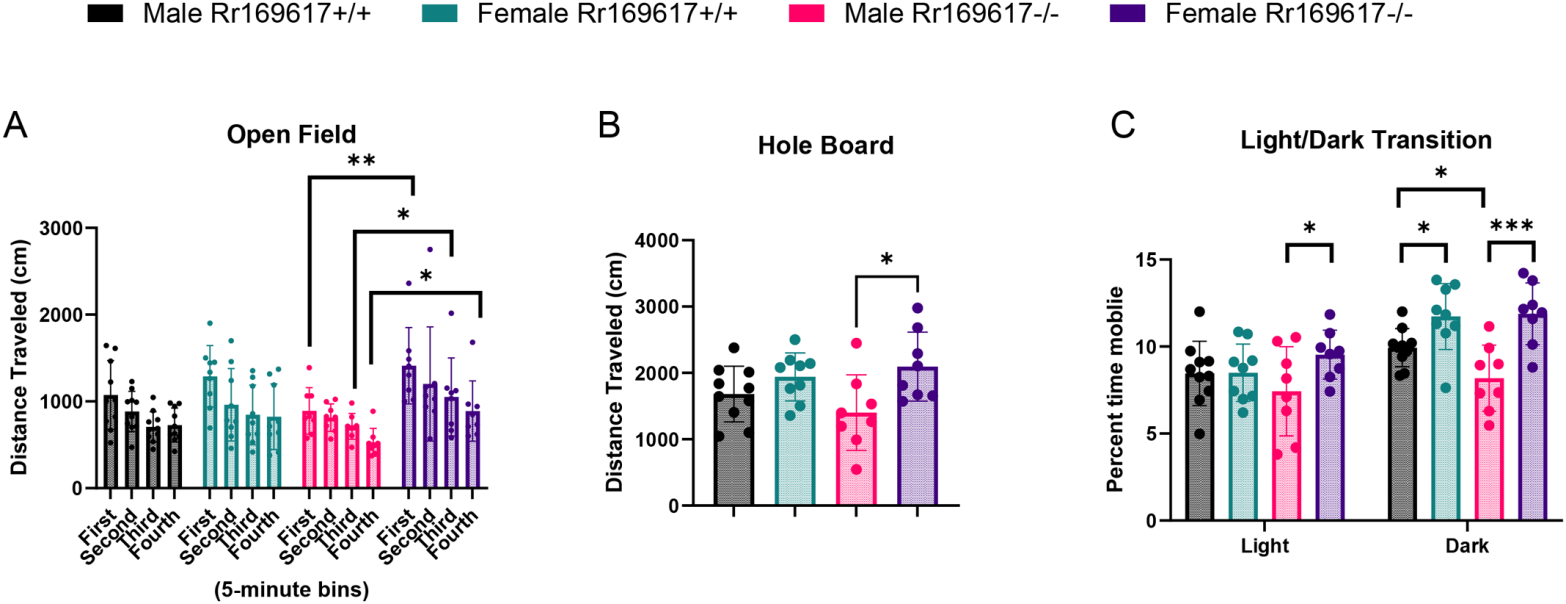
**Summary of mouse activity as measured by three independent behavioral assays.** (A) Distance travelled in 5-min bins for open field, (B) distance travelled in hole board, and (C) percentage of time mobile in light/dark transition. In all assays, male *Rr169617^−/−^* mice were less active than female *Rr169617^−/−^* mice. Asterisk indicates statistical significance (* *P*<0.05, ** *P*<0.01 *** *P*<0.001) between groups from Bonferroni corrected pairwise comparisons.

For open field, the distance travelled was recorded in four 5-min bins. There was no significant effect of gene and no overall sexual dimorphism. However, using planned pairwise comparisons, three of these bins showed sexual dimorphism within the mutant mice. Male *Rr169617^−/−^* mice travelling less than female *Rr169617^−/−^* mice (*P*-values for the first, second, third and fourth 5-min bin=0.009, 0.065, 0.031, and 0.017; Fig. 5A and Table S23). Similarly, the planned pairwise comparisons also showed sexual dimorphism within the mutant mice for time spent mobile (*P*=0.045) and time spent rearing (i.e. vertical time; *P*=0.049) (see Table S23). Overall, male *Rr169617^−/−^* mice spent less time active than female *Rr169617^−/−^* mice in open field.

The additional motor parameters recorded for hole board, revealed the same pattern, showing significance only for sexual dimorphism within the mutant mice for distance travelled (*P*=0.006; Fig. 5B), ambulatory time (*P*=0.041), and ambulatory episode average velocity (*P*=0.003) (see Table S23). Male *Rr169617^−/−^* mice travelled less distance, for less time, and more slowly than the *Rr169617^−/−^* females.

Consistent with the above findings, in the light/dark transition assay, the percentage of time mobile in the light showed male *Rr169617^−/−^* mice as less mobile than the *Rr169617^−/−^* females (*P*=0.034) (Fig. 5C). A complex pattern of statistical significance was revealed in the dark by the planned pairwise comparisons (Fig. 5C and Table S23). Both the wildtype (*P*=0.027) and the mutant mice (*P*<0.001) showed sexual dimorphism, with males consistently less mobile than females. In addition, a genotype effect for males was detected, with male *Rr169617^−/−^* mice being less mobile in the dark than their male wildtype controls (*P*=0.034). The primary driver of these differences is the reduced mobility of the male *Rr169617^−/−^* mice during light/dark transition assay (Fig. 5C).

All mice in this study were also assessed for a variety of other phenotypic measures (Table S21). Most notably, PhenStat analysis detected differences in body composition parameters that showed an overall gene effect as *Rr169617^−/−^* mice had a lower proportion of fat than wildtype mice, and conversely an increased lean proportion (e.g. fat(g)/bodyweight (g) *P*=0.005, lean mass (g)/bodyweight (g) *P*=0.002). (Table S22).

## DISCUSSION

Discovery of genes involved in autism and other neurodevelopmental disorders is occurring rapidly with the utility of several sequencing strategies. Moving beyond the exome, there is an appreciation that noncoding regions of the genome also play an important role. These regions finely tune the expression of genes and are particularly important in brain development. Several areas are being pursued with regard to noncoding regions including statistical testing for enrichment, machine-learning based models, functional characterization at thousands of regions/variants at a time (i.e. Multiplex Assays of Variant Effects), transient transgenic assays, and through precision engineering in model organisms. In this study, we follow up on our previous identification of variants in individuals with neurodevelopmental disorders in the enhancer hs737 that affects the target gene, *EBF3*. *EBF3* is well-established as a syndromic gene and has genome-wide significance for excess variation in individuals with neurodevelopmental disorders. However, the knowledge of the function of this gene and the gene regulatory network (GRN) that it resides within are understudied at this time. Our identification of hs737 provides a foothold into looking at the upstream regulators of EBF3. Further, we observed an excess of deletions of hs737 in individuals with neurodevelopmental disorders consistent with the finding that both EBF3 and its associated regulatory elements are required for normal neural development.

In this study, we pursued the hypothesis that deletion of this element in the highly conserved, orthologous mouse region would provide additional insights into hs737/Rr169617 and *EBF3*/*Ebf3*.

First, we found that when the corresponding region in mouse of hs737 is deleted it results in fewer progeny homozygous for the deletion than expected by chance. This suggests that while homozygous deletion mice are viable they are not being born at the rate expected by Mendelian inheritance. Second, mice homozygous for this deletion displayed a relatively small effect on expression of the target gene. However, each enhancer has a different effect on expression, and it is not readily apparent *a priori* what the reduction of a specific enhancer will be in an *in vivo* model. Thus, emphasizing the critical importance of experimentally testing regulatory sequences in the context of a whole animal. The modest reduction in expression can also have consequences for RNA-seq experiment design; whereby, exorbitant sample sizes would be necessary to see the expression difference as significant. Therefore, we recommend that smaller sample sizes may be pursued for the RNA-seq experiments but that the outcome of these experiments will only reveal the genes with highest changes in expression. We identified genes with high changes in expression in this study including *Lbhd1* and *Ntng1*. Third, we identified sex specific phenotypes related to mobility when comparing males versus females homozygous for the enhancer deletion. The high-throughput statistical analysis applied as standard by the IMPC indicated greater exploration by female mutants compared to wildtype and less exploration by male mutants compared to wildtype. A smaller but very consistent sexually dimorphic effect, specific to the mutants, was seen across three independently conducted behavioral assays, with male *Rr169617^−/−^* mice less mobile than the *Rr169617^−/−^* females (nine of ten additional significant motor measures assessed by planned pairwise comparisons). This is highly relevant to the phenotype of autism and the phenotypes we see in individuals with variation in the hs737 enhancer (males with autism and hypotonia but no intellectual disability) (Padhi et al., 2021).

In future studies, it will be important to identify the upstream transcription factors that bind hs737, determine its activity at the single-cell level, and characterize the other cis-regulatory elements that help orchestrate the precise developmental expression of *Ebf3*. These analyses will provide a framework for investigating the effects of other noncoding variants found within the *Ebf3* regulatory landscape. Here, we focused on the deletion of a single element due to the overwhelming supporting evidence from individuals with neurodevelopmental disorders that harbor deletions in this region. The detailed characterization of this novel mouse model provides insight into the molecular and phenotypic impacts of deleting this enhancer and will be of interest to the broad biomedical research community interested in understanding how changes in the noncoding fraction of the genome affect human health and disease.

## MATERIALS AND METHODS

### Generation of deletion mouse lines

To delete the relevant sequence in Rr169617, paired upstream (CATGCAGAGAAAACAAAATG, GCTGAATTGTAGCGTGTTTA) and downstream (TGGCGCCAGTGGGCCCCGAC, ATCCTGGCACTGGCGCCAGT) guides were identified to flank the genomic region of interest on mouse chr7:136083335-136084349 (GRCm38/mm10). Guide RNAs were incubated with Cas9 protein to generate ribonucleoprotein complexes (RNPs) followed by electroporation into C57BL/6J zygotes (JAX strain #:000664) using standard conditions. Following PCR genotyping for the deletion allele three independent founder lines (lines 299, 300 and 304) were recovered and backcrossed to C57BL/6J to generate N1 progeny; however, only two lines (299 and 304) showed successful germline transmission. A molecular description of the genomic lesion present in each of these independent lines was defined by Sanger Sequencing of PCR amplicons. Line 299 carried a 1160 bp deletion (chr7:136083275-136084434 GRCm38/mm10); referred to as C57BL/6J-*Rr169617^em1Tnt^*/J (RRID:MMRRC_075665-MU). Line 304 was found to contain a 1147 bp deletion (chr7:136083283-136084428 GRCm38/mm10); referred to as C57BL/6J-*Rr169617^em2Tnt^*/J. In this study, detailed characterization was performed on C57BL/6J-*Rr169617^em1Tnt^*/J that we refer to as Rr169617 in this study.

### Ethical approval

All mouse work reported herein was conducted at the Jackson Laboratory under the Institutional Animal Care and Use Committee-approved license numbers 11005 and 20028. AAALACi accreditation number 00096, and NIH Office of Laboratory Animal Welfare assurance number D16-00170.

### Animal housing information

Animals used in phenotyping studies were homozygous mutant *Rr169617^−/−^* mice [female (*n*=8), male (*n*=8)] and age and sex matched wildtype control *Rr169617^+/+^* mice [female (*n*=9), male (*n*=10)]. Mice were housed (1 to 5 animals per cage) in individually ventilated cages [Thoren Duplex II Mouse Cage #11 and Thoren Maxi-Miser PIV System (30.8 L×30.8 W×16.2 H cm)] behind a pathogen-free barrier. Access to water and food (5K52 diet, LabDiet) was *ad libitum*. Wood shavings (aspen) bedding substrate was provided and sections housing individual mice were supplemented with environmental enrichments (e.g. a nestlet and cardboard hut). Mice were housed in rooms with 12-h light–dark cycle and temperature and humidity were maintained between 20-22°C and 44-60%, respectively.

### Mouse colony maintenance and embryo collections

Mouse colonies were maintained by either backcrossing to wildtype C57BL/6J or by intercrosses between heterozygous animals. Timed matings were performed by intercrossing heterozygous *Rr169617^+/−^* animals where noon of the day of detection of vaginal plug was considered embryonic day 0.5 (E0.5). Embryos were kept cold on ice in 1× phosphate buffered saline (PBS) and microdissected in ice cold PBS. Embryonic tissues were snap frozen in liquid nitrogen and stored at −80°C until use.

### Genotyping PCR for the deletion

DNA was extracted from E12.5 forebrains for one sample each of wildtype (*Rr169617^+/+^*), heterozygous (*Rr169617^+/−^*), and homozygous (*Rr169617^−/−^*) mice using the Zymo Quick-DNA HMW MagBead kit. This extraction method was also used to derive DNA from the HT-22 cell line as a control for the PCR. Primers were designed to test for presence of the deletion in Rr169617 (mm10 chr7:136083275-136084434). The forward primer was 5′ CATACTTAGCTACTGTGGATGGTGA 3′ and the reverse primer was 5′ CAAATCCCACCTTAACAGCACATAG 3′. PCR reactions consisted of 30 ng HMW DNA of each sample, positive control (HT-22), or negative control (water), 1.25 µl of 10 µM forward primer, 1.25 µl of 10 µM reverse primer, 0.75 µl DMSO, 12.5 µl 2× Phusion High Fidelity master mix, and nuclease free water up to 25 µl. Cycling conditions were 98°C for 2 min, 25 cycles of [98°C for 10 s, 70°C for 30 s, 72°C for 30 s], 72°C for 10 min, 4°C hold. The samples were run on an Agilent Bioanalyzer. The wildtype band was 2618 bp and the deletion-containing band was 1484 bp. Confirmation of the sequence of the wildtype and deletion bands were completed by TOPO TA cloning of the sequences into a plasmid (using the TOPO TA Cloning Kit for Sequencing) and sequencing of the plasmid by Oxford Nanopore Technology sequencing at Plasmidsaurus.

### Long-read whole-genome sequencing of mice

E12.5 mouse forebrains were pooled from three wildtype (*Rr169617^+/+^*) and three homozygous mice (*Rr169617^−/−^*), respectively. High molecular weight DNA was extracted for each pooled sample. Each pool was made into a library for PacBio HiFi sequencing on the Revio sequencer. Each library was sequenced using one SMRT cell to approximately 30× coverage.

### RNA extraction, cDNA synthesis and RNA-seq of E12.5 forebrain

Mouse E12.5 fetal forebrain tissue from mice that were wildtype (*Rr169617^+/+^*) (*n*=12), heterozygous (*Rr169617^+/−^*) (*n*=14), or homozygous (*Rr169617^−/−^*) (*n*=10) for the deletion in Rr169617 was used to extract RNA. The RNA was extracted using a Bead Bug homogenizer to homogenate the tissue and the Maxwell simplyRNA Tissue kit for RNA extraction. SuperScript III First-Strand Synthesis System was used for reverse transcription. Taqman mouse *Ebf3* (Mm00438642_m1) and GAPDH (Mm99999915_g1) gene expression assays were performed on a QuantStudio 6 Flex quantitative thermocycler using four reactions for each sample. QuantStudio Real-Time PCR software was used to run the thermocycler using the Standard Comparative Ct (ΔΔCt) method. Three individuals performed a total of five qPCR assays in quadruplicate. Results were reviewed by three individuals to assess each set of quadruplicates for outliers (>0.5 cycles apart), and these were removed from the data sets. For RNA-seq, three RNA samples from each E12.5 genotype group (*Rr169617^+/+^*, *Rr169617^+/−^*, *Rr169617^−/−^*) were polyA selected and sequenced to a target of 200 million read pairs using Illumina NovaSeq6000. Every sample RNA had a RIN greater than 8.0. Ribosomal RNA was removed through poly-A selection with Oligo-dT beads (mRNA Direct kit, Life Technologies). The mRNA was fragmented in reverse transcriptase buffer and heated to 94°C for 8 min. Reverse transcription of the mRNA to cDNA was performed using the SuperScript III RT enzyme with random hexamers. A second strand synthesis was carried out to produce double-stranded cDNA. The cDNA was blunt-ended, an A base was added to the 3′ ends, and Illumina sequencing adapters were ligated to the ends. The ligated fragments were amplified for 12-15 cycles with primers incorporating unique dual index tags. Finally, the fragments were sequenced on an Illumina NovaSeq with paired-end reads extending 150 bases at the McDonnell Genome Institute.

### RNA-seq of E12.5 midbrain and hindbrain

RNA was extracted from E12.5 midbrains and hindbrains of three independent samples of each genotype (*Rr169617^+/+^*, *Rr169617^−/−^*), respectively. Library preparation, ribosomal RNA reduction, and Illumina UDI library preparation were performed at the University of Maryland Institute for Genome Sciences. They were sequenced (rRNA depletion RNA-seq) to a target of 200 million read pairs using an Illumina NovaSeq6000.

### RNA-seq analysis

The RNA-seq analysis was run using the ENCODE pipeline, found here (https://github.com/ENCODE-DCC/rna-seq-pipeline), due to it being a well-developed standard. The only modifications were hardcoded PATH variables so that the pipeline would function properly on our HPC. The mouse Gencode M21 reference data was used; links are provided in the ENCODE documentation. The forebrain poly-A samples were run as paired, unstranded runs, while the rRNA-depleted samples were run as paired, reverse-stranded runs. Differential gene expression analysis was performed using DESeq2 (Love et al., 2014).

### Phenotype pipeline

The mice progressed through the JAX KOMP phenotyping pipeline (Table S5, https://www.mousephenotype.org/impress/PipelineInfo?id=12) (Dickinson et al., 2016). The methods for all assays in the pipeline are provided online (https://www.mousephenotype.org/impress/PipelineInfo?id=12) and the assays for which we had *a priori* hypotheses are detailed below. Raw phenotyping data are available in Tables S4-S20.

### Behavioral assays

Three behavioral assays (open field, light/dark transition and hole board) were conducted to provide information on anxiety, exploration and mobility. Testing was conducted between 07:00 h and 17:00 h in the light portion of their 24-h cycle and mice were first habituated to the room for 30 min. For all three assays, mice were placed in an acrylic chamber (40×40×40 cm) contained within a sound attenuated, ventilated cabinet (64 L×60 W×60 H cm) and the motor behavior and location were recorded by horizontal infrared photobeam sensors (16×16 array) using Fusion behavioral tracking software (Omnitech Electronics, Columbus, OH, USA).

At approximately 8 weeks of age mice completed the open field test. Mice were placed in the center of the open field arena (light level:100-200 lux) and behavior was recorded for 20 min. The following week mice were tested in the light/dark transition assay which included a dark insert chamber (40×20×40 cm) so that half the chamber was in light (∼200 lux) and half dark (∼1 lux). Mice were placed in the lit portion of the chamber facing away from the dark portion and were recorded for 20 min. Later the same week mice were tested in the hole board configuration which included a grid of 16 shallow holes in the floor (4×4 grid) of the open field arena for the mice to explore (light level:100-200 lux) and behavior was recorded for 10 min.

### Dual-energy X-ray absorptiometry (DEXA)

DEXA provided measures of body composition (lean and fat mass), bone mineral content and bone density. Mice (approximately 14 weeks of age) were anesthetized [intraperitoneal injection of 400 mg/kg tribromoethanol diluted in sterile PBS (in-house pharmacy)], measured for length and then placed in the previously calibrated densitometry machine (Lunar Piximus II from GE Medical systems). The region of interest measured excluded the head and neck.

### Statistical analysis of phenotypes

The phenotyping pipeline was analyzed using standard IMPC analysis based on PhenStat (Kurbatova et al., 2015) which was designed to find the best analysis for high-throughput data. Standard mandatory parameters for the IMPC were analyzed (Table S21; https://www.mousephenotype.org/impress/PipelineInfo?id=12). Continuous data were analyzed using optimized linear model ANOVAs with the initial factors of genotype, sex, and body weight when available. PhenStat returns significance and effect size for genotype and for sexual dimorphism. Categorical data (e.g. eye morphology) were tested using Fisher's exact tests.

Targeted *a priori* hypotheses: Although the mice were tested in a full phenotyping pipeline, several of the assays provide additional, non-mandatory parameters of motor responses that were hypothesized to differ between the sexes of the mutant mice (Padhi et al., 2021). These additional behavioral data were analyzed using multivariate ANOVAs by assay, testing factors of sex (male, female), genotype (*Rr169617^−/−^*, *Rr169617^+/+^*), and interaction between sex and genotype. Mutant male mice were predicted to be more affected than mutant female mice (Padhi et al., 2021) therefore planned paired comparisons of sex by genotype were completed for these assays, with Bonferroni adjustment for multiple testing, for each analysis. These *a priori* hypotheses were analyzed using SPSS version 29 (IBM). Motor parameters previously tested using PhenStat were not re-analyzed.

## Supplementary Material

10.1242/biolopen.062070_sup1 Supplementary information

Table S1. E12.5 Forebrain RNAseq Results

Table S2. E12.5 Midbrain RNAseq Results

Table S3. E12.5 Hindbrain RNAseq Results

Table S4. Mouse Details for the KOMP Phenotyping

Table S5. KOMP Phenotyping Pipeline

Table S6. KOMP Phenotyping Standalone Body Weight

Table S7. KOMP Phenotyping Open Field

Table S8. KOMP Phenotyping SHIRPA Dysmorphology

Table S9. KOMP Phenotyping Grip Strength

Table S10. KOMP Phenotyping Light/Dark Transition

Table S11. KOMP Phenotyping Hole Board

Table S12. KOMP Phenotyping Acoustic Startle PPI

Table S13. KOMP Phenotyping Electrocardiography

Table S14. KOMP Phenotyping Glucose Tolerance

Table S15. KOMP Phenotyping Body Composition

Table S16. KOMP Phenotyping Eye Morphology

Table S17. KOMP Phenotyping Auditory Brainstem Response

Table S18. KOMP Phenotyping Hematology

Table S19. KOMP Phenotyping Clinical Blood Chemistry

Table S20. KOMP Phenotyping Heart Weight

Table S21. Phenotyping Full Parameter List

Table S22. Phenstat Significant Results

Table S23. ANOVA with Planned Pairwise Comparisons (PPC) Significant
