# Supplementary material for: Generation and characterization of a knockout mouse of an enhancer of EBF3
Source: Biol Open. 2025 Nov 7;14(11):bio062070. doi: 10.1242/bio.062070 (PMC12641488; doi:10.1242/bio.062070)
Supplement: Supplementary information [file biolopen-14-062070-s1.pdf]

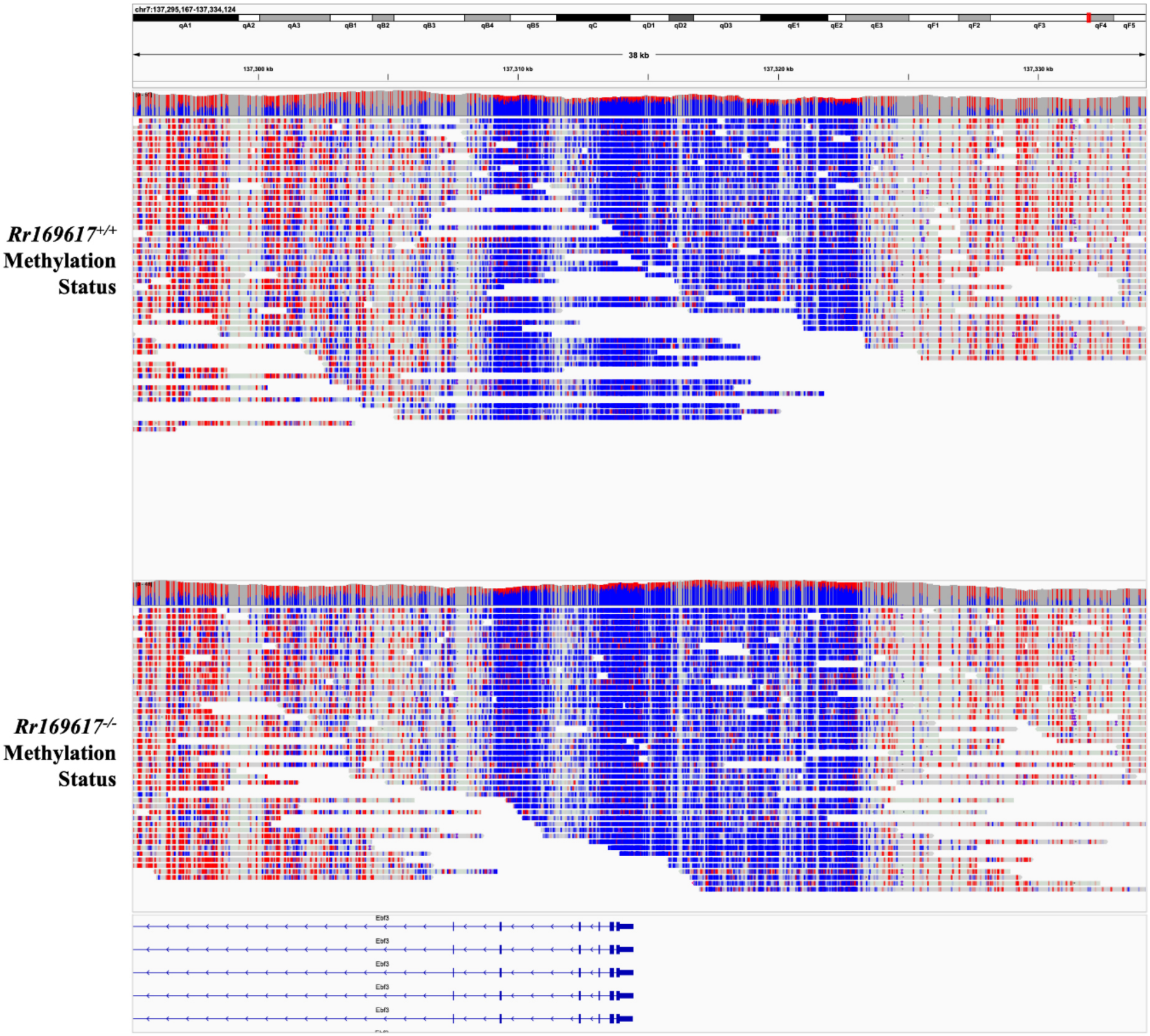

Fig. S1. Methylation Status at the *Ebf3* Promoter in *Rr169617*$^{+/+}$ and *Rr169617*$^{-/-}$ E12.5 forebrains. Shown is the methylation status of CpG sites within the *Ebf3* promoter region based on the PacBio whole-genome sequencing data. The methylation patterns look similar in both. Red = methylated CpG. Blue = unmethylated CpG.

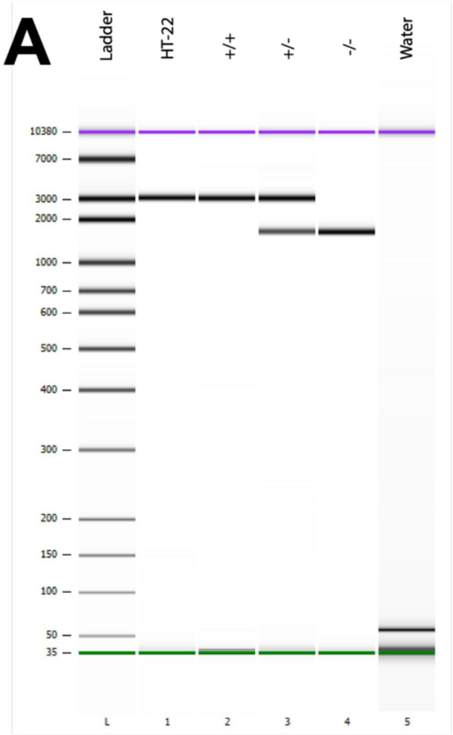

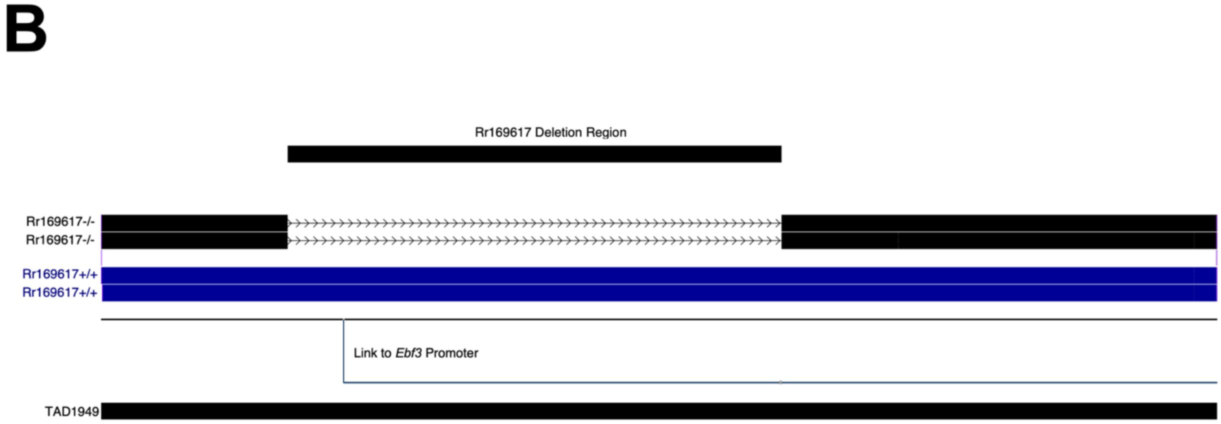

Fig. S2. Genotyping assay for 1,160 bp deletion in Rr169617 mice. A) Results of PCR-based assay to genotype for the deletion. B) Sequencing of PCR products confirming they match the *Rr169617*$^{+/+}$ and *Rr169617*$^{-/-}$ expected results. Shown are two replicates of each.

Biology Open • Supplementary information

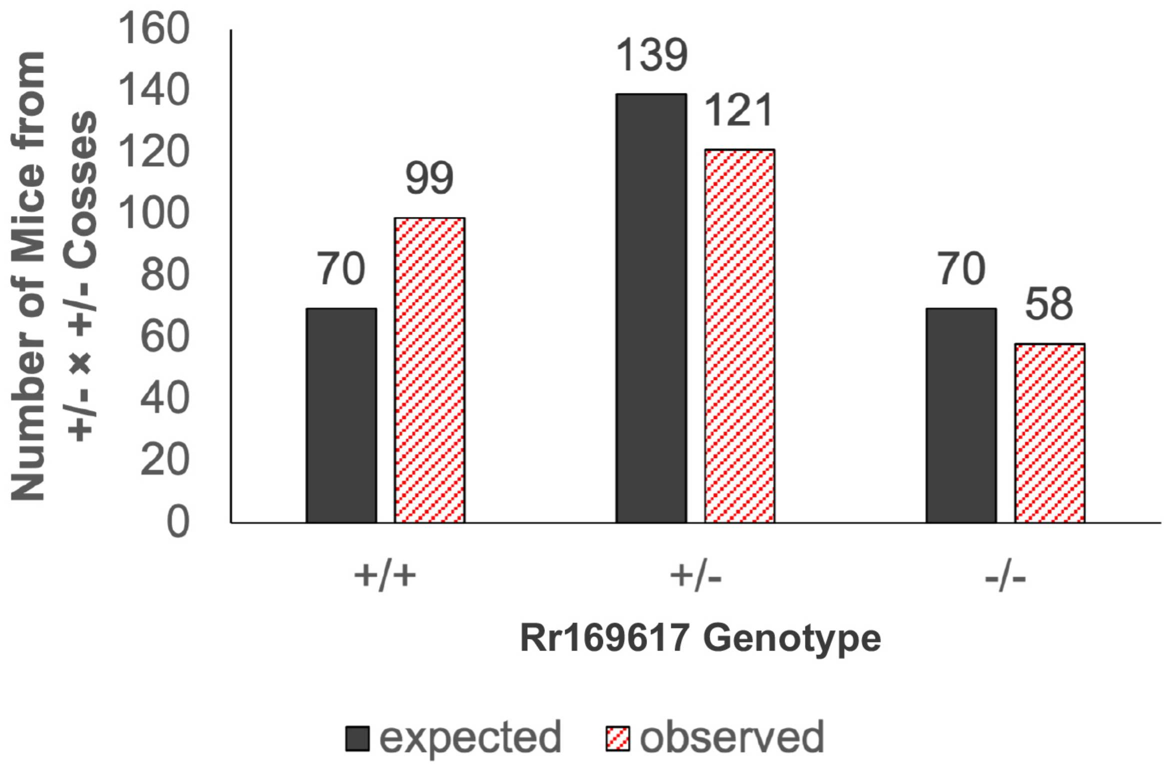

Fig. S3. Genotypes of 278 Mice from *Rr169617*$^{+/-}$ × *Rr169617*$^{+/-}$ Crosses.
Shown in black are the expected counts (based on Mendelian inheritance) and in red are the actual observed counts from heterozygote crosses. This distribution significantly deviates from expected Mendelian frequencies (Chi-Square Test, $p = 0.02$). Genotyping of the mice assessed in this figure was performed at the time of weaning.

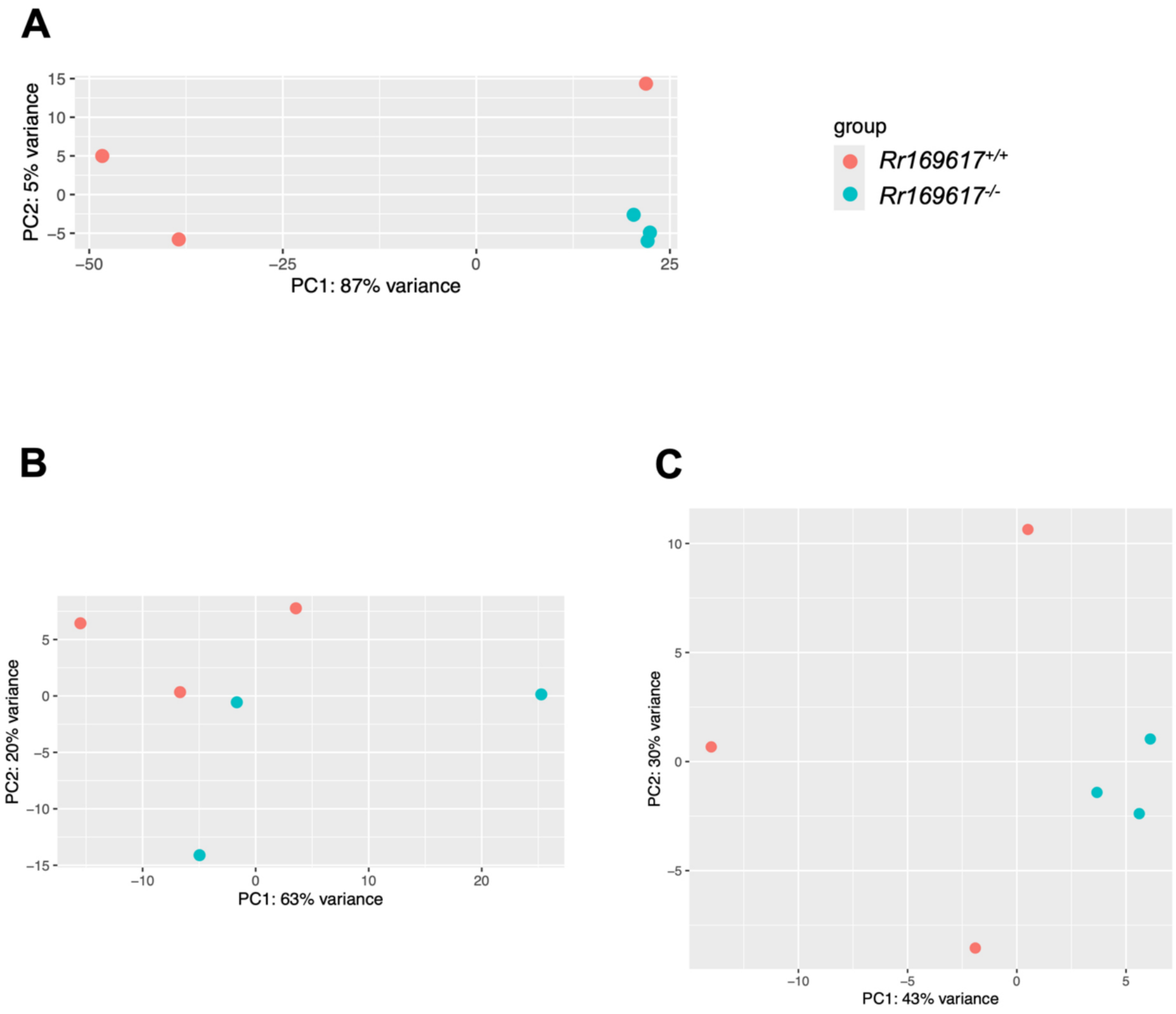

Fig. S4. PCA plots of RNA-seq data from *E12.5 Rr169617*$^{-/-}$ and *Rr169617*$^{+/+}$ mice. PCA plots showing the clustering of the wildtype and homozygous deletion samples in A) forebrain, B) rRNA-depleted midbrain, and C) rRNA-depleted hindbrain mice collected at E12.5.

Biology Open • Supplementary information

Table S1. E12.5 Forebrain RNAseq Results

Available for download at
https://journals.biologists.com/bio/article-lookup/doi/10.1242/bio.062070#supplementary-data

Table S2. E12.5 Midbrain RNAseq Results

Available for download at
https://journals.biologists.com/bio/article-lookup/doi/10.1242/bio.062070#supplementary-data

Table S3. E12.5 Hindbrain RNAseq Results

Available for download at
https://journals.biologists.com/bio/article-lookup/doi/10.1242/bio.062070#supplementary-data

Table S4. Mouse Details for the KOMP Phenotyping

Available for download at
https://journals.biologists.com/bio/article-lookup/doi/10.1242/bio.062070#supplementary-data

Table S5. KOMP Phenotyping Pipeline

Available for download at
https://journals.biologists.com/bio/article-lookup/doi/10.1242/bio.062070#supplementary-data

Table S6. KOMP Phenotyping Standalone Body Weight

Available for download at
https://journals.biologists.com/bio/article-lookup/doi/10.1242/bio.062070#supplementary-data

Table S7. KOMP Phenotyping Open Field

Available for download at
https://journals.biologists.com/bio/article-lookup/doi/10.1242/bio.062070#supplementary-data

Table S8. KOMP Phenotyping SHIRPA Dysmorphology

Available for download at
https://journals.biologists.com/bio/article-lookup/doi/10.1242/bio.062070#supplementary-data

Table S9. KOMP Phenotyping Grip Strength

Available for download at
https://journals.biologists.com/bio/article-lookup/doi/10.1242/bio.062070#supplementary-data

Table S10. KOMP Phenotyping Light/Dark Transition

Available for download at
https://journals.biologists.com/bio/article-lookup/doi/10.1242/bio.062070#supplementary-data

Table S11. KOMP Phenotyping Hole Board

Available for download at
https://journals.biologists.com/bio/article-lookup/doi/10.1242/bio.062070#supplementary-data

Table S12. KOMP Phenotyping Acoustic Startle PPI

Available for download at
https://journals.biologists.com/bio/article-lookup/doi/10.1242/bio.062070#supplementary-data

Table S13. KOMP Phenotyping Electrocardiography

Available for download at
https://journals.biologists.com/bio/article-lookup/doi/10.1242/bio.062070#supplementary-data

Table S14. KOMP Phenotyping Glucose Tolerance

Available for download at
https://journals.biologists.com/bio/article-lookup/doi/10.1242/bio.062070#supplementary-data

Table S15. KOMP Phenotyping Body Composition

Available for download at
https://journals.biologists.com/bio/article-lookup/doi/10.1242/bio.062070#supplementary-data

Table S16. KOMP Phenotyping Eye Morphology

Available for download at
https://journals.biologists.com/bio/article-lookup/doi/10.1242/bio.062070#supplementary-data

Table S17. KOMP Phenotyping Auditory Brainstem Response

Available for download at
https://journals.biologists.com/bio/article-lookup/doi/10.1242/bio.062070#supplementary-data

Table S18. KOMP Phenotyping Hematology

Available for download at
https://journals.biologists.com/bio/article-lookup/doi/10.1242/bio.062070#supplementary-data

Table S19. KOMP Phenotyping Clinical Blood Chemistry

Available for download at
https://journals.biologists.com/bio/article-lookup/doi/10.1242/bio.062070#supplementary-data

Table S20. KOMP Phenotyping Heart Weight

Available for download at
https://journals.biologists.com/bio/article-lookup/doi/10.1242/bio.062070#supplementary-data

Table S21. Phenotyping Full Parameter List

Available for download at
https://journals.biologists.com/bio/article-lookup/doi/10.1242/bio.062070#supplementary-data

Table S22. Phenstat Significant Results

Available for download at
https://journals.biologists.com/bio/article-lookup/doi/10.1242/bio.062070#supplementary-data

Table S23. ANOVA with Planned Pairwise Comparisons (PPC) Significant

Available for download at
https://journals.biologists.com/bio/article-lookup/doi/10.1242/bio.062070#supplementary-data

Biology Open • Supplementary information
